# Flomoxef for neonates: extending options for treatment of neonatal sepsis caused by ESBL-producing Enterobacterales—authors’ response

**DOI:** 10.1093/jac/dkac072

**Published:** 2022-03-04

**Authors:** Christopher A Darlow, William Hope

**Affiliations:** Antimicrobial Pharmacodynamics and Therapeutics, University of Liverpool, Liverpool, UK; Antimicrobial Pharmacodynamics and Therapeutics, University of Liverpool, Liverpool, UK

We thank Joseph F. Standing for his comments^1^ regarding our recently published population pharmacokinetic model for flomoxef in neonatal populations aged <4 weeks.^2^

To briefly summarize our approach: we fitted a population model to the flomoxef pharmacokinetic data (available in the public domain) for infants predominantly aged <28 days. The fitting of a base model to the data (i.e. without the inclusion of covariates such as age and weight) somewhat surprisingly showed an exponential rise in clearance in the first 28 days of life. This observation is accounted for by a combination of changes in size (allometry) and development (ontogeny). We could have estimated the scaling exponent that relates size (weight) to clearance, but we would have constrained the potential value(s) to <1 to avoid violating well-characterized allometric relationships that are operational in a wide range of biological contexts. Ultimately, we chose to fix this value at 0.75. We attributed the additional changes in clearance with increasing age and weight to time- and gestation-dependent maturation in renal function, with the subsequent scalars of these parameters estimated from the data. We note that significant (but, as yet, uncharacterized) drug transporters, with their own idiosyncratic ontogeny functions,^3^ rather than passive glomerular filtration alone, likely account for a large proportion of the total flomoxef clearance.^4^ This insight highlights the danger of mechanistically linking clearance to glomerular filtration rate.

While we agree the overall shape of the clearance versus weight and age curves are ultimately sigmoidal with the upper limit approximating adult values, we had no data to estimate the transition of clearance from neonates to children and adults. We could have constrained the solution and/or used a different structural model, but, in the absence of data, we did not feel this was appropriate. A consequence of that decision is that the model only describes the pharmacokinetics of flomoxef in neonates aged <28 days.

Our population pharmacokinetic model enabled us to describe the prolonged gamma phase of flomoxef even in heavier and older infants. The Monte Carlo simulations were well-centred and recapitulated the original population, including older heavier infants. We note the series of figures that accompany the letter by Standing,^1^ but we do not believe they are reflective of the time–concentration profiles predicted by our model (Figure 1). Our own approach is to apply considerable caution in making predictions from pharmacokinetic models to populations other than those for which they are developed, and this is no exception to that rule. We confirm that (for neonates aged <28 days) the candidate regimens and PTA analyses we report are robust, with findings that are congruent with those described in the flomoxef Summary of Product Characteristics,^5^ and we continue to suggest that flomoxef is potentially an extremely valuable agent for the treatment of neonatal sepsis.

**Figure 1.**
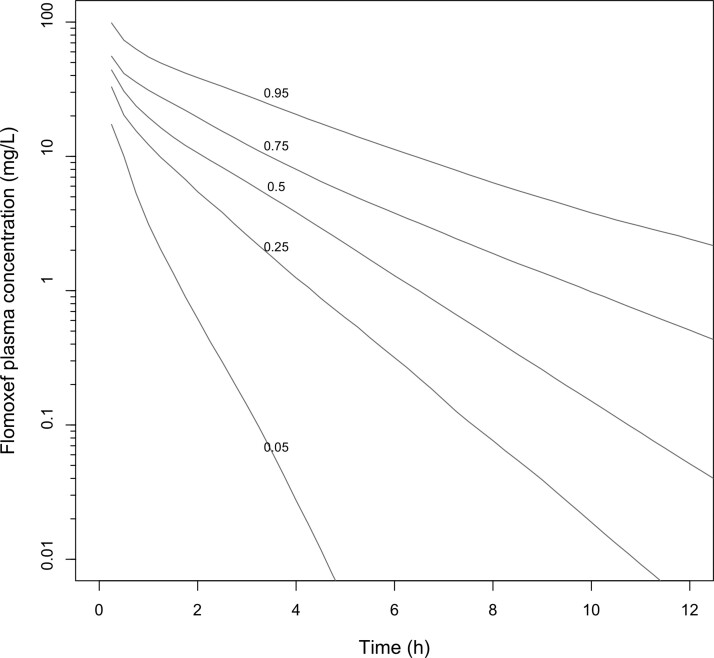
Monte Carlo simulation of flomoxef drug exposures following a single 20 mg/kg IV dose of flomoxef in 10 000 neonates aged 21–28 days, weighing 2–4 kg, with time–concentration profiles for the 5th, 25th, 50th, 75th and 95th centiles of the simulated population.

## Transparency declarations

Shionogi have provided flomoxef material free of charge for other experimental work conducted by the authors, but had no input into this work. C.A.D. has nothing further to declare. W.H. holds or has recently held research grants with UKRI, F2G, Spero Therapeutics, Antabio, Pfizer, Bugworks, Phico Therapeutics, BioVersys, GARDP and NAEJA-RGM. He is (or has recently been) a consultant for Appili Therapeutics, F2G, Spero Therapeutics, NAEJA-RGM, Centauri, Pfizer, Phico Therapeutics and VenatoRx. He is a member of the Specialist Advisory Committee for GARDP and the Specialty National Co-lead for Infectious Diseases for the National Institute of Health Research (NIHR).
